# Oxytocin-Dependent Regulation of TRPs Expression in Trigeminal Ganglion Neurons Attenuates Orofacial Neuropathic Pain following Infraorbital Nerve Injury in Rats

**DOI:** 10.3390/ijms21239173

**Published:** 2020-12-01

**Authors:** Masatoshi Ando, Yoshinori Hayashi, Suzuro Hitomi, Ikuko Shibuta, Akihiko Furukawa, Tatsuki Oto, Takanobu Inada, Tomoyuki Matsui, Chikashi Fukaya, Noboru Noma, Masakazu Okubo, Yoshiyuki Yonehara, Tadayoshi Kaneko, Koichi Iwata, Masamichi Shinoda

**Affiliations:** 1 Department of Oral and Maxillofacial Surgery, Nihon University School of Dentistry, Tokyo 101-8310, Japan; dema17002@g.nihon-u.ac.jp (M.A.); furukawa.akihiko@nihon-u.ac.jp (A.F.); yonehara.yoshiyuki@nihon-u.ac.jp (Y.Y.); kaneko.tadayoshi@nihon-u.ac.jp (T.K.); 2 Department of Physiology, Nihon University School of Dentistry, Tokyo 101-8310, Japan; hayashi.yoshinori@nihon-u.ac.jp (Y.H.); hitomi.suzuro@nihon-u.ac.jp (S.H.); shibuta.ikuko@nihon-u.ac.jp (I.S.); iwata.kouichi@nihon-u.ac.jp (K.I.); 3 Department of Complete Denture Prosthodontics, Nihon University School of Dentistry, Tokyo 101-8310, Japan; deta18004@g.nihon-u.ac.jp; 4 Department of Oral and Maxillofacial Surgery, Showa University School of Dentistry, Tokyo 142-8555, Japan; inada-t-5@dent.showa-u.ac.jp; 5 Department of Pediatric Dentistry, Nihon University School of Dentistry, Tokyo 101-8310, Japan; deto17027@g.nihon-u.ac.jp; 6 Division of Applied System Neuroscience, Department of Neurological Surgery, Nihon University School of Medicine, Tokyo 173-8610, Japan; fukaya.chikashi@nihon-u.ac.jp; 7 Department of Oral Diagnostic Sciences, Nihon University School of Dentistry Tokyo 101-8310, Japan; noma.noboru@nihon-u.ac.jp; 8 Department of Removable Prosthodontics, Nihon University School of Dentistry at Matsudo, Matsudo 271-8587, Japan; okubo.masakazu@nihon-u.ac.jp

**Keywords:** oxytocin, TRPV1, TRPV4, infraorbital nerve injury, orofacial mechanical allodynia

## Abstract

We evaluated the mechanisms underlying the oxytocin (OXT)-induced analgesic effect on orofacial neuropathic pain following infraorbital nerve injury (IONI). IONI was established through tight ligation of one-third of the infraorbital nerve thickness. Subsequently, the head withdrawal threshold for mechanical stimulation (MHWT) of the whisker pad skin was measured using a von Frey filament. Trigeminal ganglion (TG) neurons innervating the whisker pad skin were identified using a retrograde labeling technique. OXT receptor-immunoreactive (IR), transient receptor potential vanilloid 1 (TRPV1)-IR, and TRPV4-IR TG neurons innervating the whisker pad skin were examined on post-IONI day 5. The MHWT remarkably decreased from post-IONI day 1 onward. OXT application to the nerve-injured site attenuated the decrease in MHWT from day 5 onward. TRPV1 or TRPV4 antagonism significantly suppressed the decrement of MHWT following IONI. OXT receptors were expressed in the uninjured and Fluoro-Gold (FG)-labeled TG neurons. Furthermore, there was an increase in the number of FG-labeled TRPV1-IR and TRPV4-IR TG neurons, which was inhibited by administering OXT. This inhibition was suppressed by co-administration with an OXT receptor antagonist. These findings suggest that OXT application inhibits the increase in TRPV1-IR and TRPV4-IR TG neurons innervating the whisker pad skin, which attenuates post-IONI orofacial mechanical allodynia.

## 1. Introduction

Orofacial neuropathic pain is caused by trigeminal nerve injury, which results from tooth extraction, maxillary bone fracture, or dental implant displacement [1,2]. Although patients with orofacial neuropathic pain caused by trigeminal nerve injury present prolonged and intractable orofacial pain hypersensitivities, there remains no established treatment for orofacial neuropathic pain since the mechanisms underlying pain hypersensitivities remain unclear [3].

Oxytocin (OXT) is a well-known hormone that is synthesized by neurosecretory cells in the paraventricular nucleus and supraoptic nucleus of the hypothalamus; moreover, it is secreted from the posterior pituitary gland. It is involved in the contraction of uterine smooth muscles and muscle fibers of the mammary gland to promote lactation [4]. Furthermore, it has been reported that intrathecal or systemic OXT administration exerts analgesic effects by binding to the OXT receptor [5,6,7]. Primary sensory neurons express OXT receptors and their excitation is inhibited by peripheral OXT signaling via the OXT receptor [8,9]. Recently, OXT receptor was identified in the sensory neurons of the trigeminal ganglion (TG), while orofacial inflammation was identified to cause an upregulation of OXT receptor expression in the TG neurons [10]. These findings suggest that OXT signaling in the TG neurons via the OXT receptor may modulate TG neuronal excitability, which is closely related to orofacial pain sensitivity.

Transient receptor potential (TRP) channels are expressed in the primary sensory neurons and are involved in numerous biological functions, especially in the regulation of pain sensation [11,12]. In the TRP channel family, although TRP vanilloid 1 (TRPV1) is predominantly expressed in the sensory neurons, TRPV4 is expressed at multiple sites, mostly in the kidneys, internal organs, and nociceptive neurons of the TG, among others [13]. TRPV1 is expressed only in small and medium-sized TG neurons, which are assumed to be nociceptors and are activated by peripheral capsaicin, peripheral noxious heat (>43 °C), and protons in rodents [11,14]. TRPV4 is activated by warm temperature (>27 °C) and extracellular osmolarity, indicating its role as a mechanosensitive channel [15]. Peripheral TRPV4 antagonism suppresses xerostomia-induced tongue mechanical allodynia in rodents [16]. In some studies, changes in the TRPV1 and TRPV4 expression in the primary nociceptive neurons following trigeminal nerve injury or orofacial trauma were reported to be involved in orofacial pain hypersensitivity [17,18,19]. Furthermore, the OXT receptor is localized in the primary nociceptive neurons [8]. These reports suggest that OXT signaling via the OXT receptor is involved in changes in the functional characteristics of TRPV1 and TRPV4, which are associated with orofacial pain sensitivity.

This study aimed to evaluate the involvement of OXT signaling via OXT receptors in changes in the functional characteristics of TRPV1 and TRPV4 associated with orofacial neuropathic pain following infraorbital nerve (ION) injury (IONI).

## 2. Results

### 2.1. Effect of Local OXT Administration on Post-IONI Orofacial Pain Hypersensitivity

**Figure 1:**
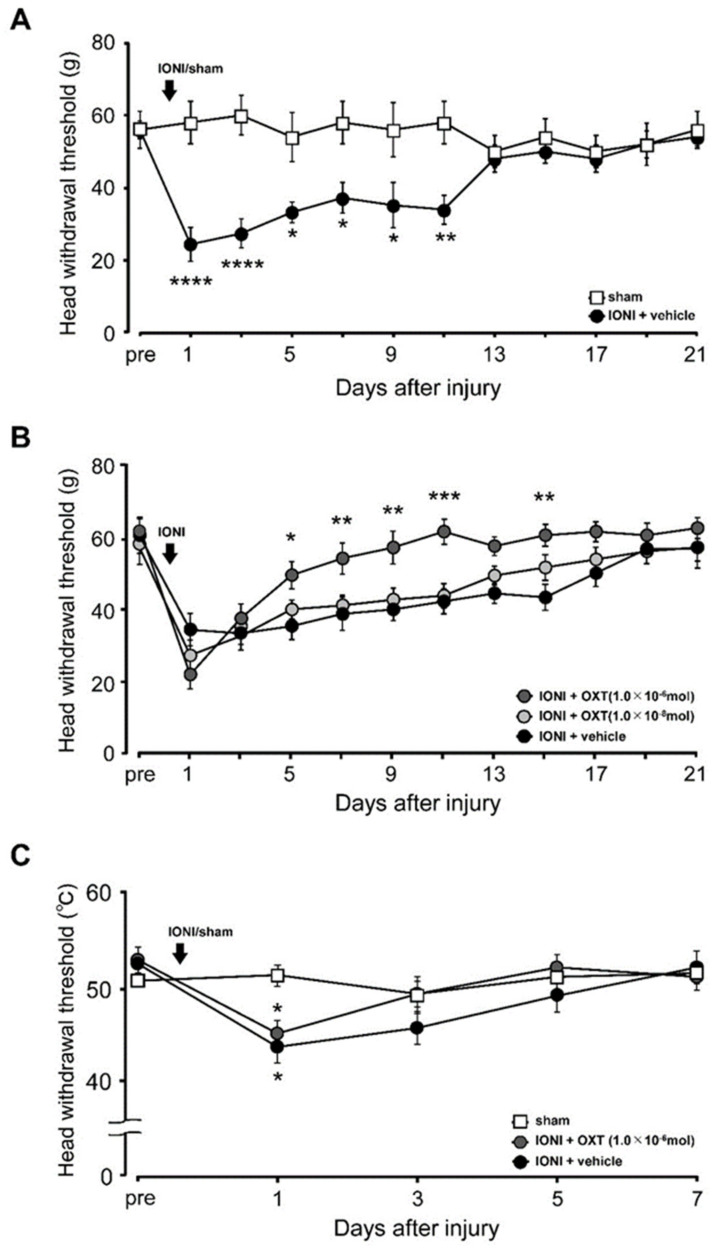
Changes in mechanical and heat sensitivities of the whisker pad skin after infraorbital nerve injury (IONI) or sham treatment. (**A**) The head withdrawal threshold for mechanical stimulation (MHWT) of the whisker pad skin after IONI or sham treatment (*n* = 15 in each group). * *p* < 0.05, ** *p* < 0.01, **** *p* < 0.0001 (vs. the sham treatment group). (**B**) The MHWT of the whisker pad skin after IONI with vehicle or oxytocin (OXT) administration (IONI + OXT (1.0 × 10^−6^ mol), *n* = 10; IONI + OXT (1.0 × 10^−8^ mol), *n* = 9; IONI + vehicle, *n* = 9). * *p* < 0.05, ** *p* < 0.01, *** *p* < 0.001 (vs. the IONI treatment with vehicle group). (**C**) The heat head withdrawal threshold of the whisker pad skin after sham treatment or IONI with vehicle or OXT administration (sham, *n* = 5; IONI + OXT (1.0 × 10^−6^ mol), *n* = 10; IONI + vehicle, *n* = 10). * *p* < 0.05, (vs. the sham treatment group).

There was a significant decrease in the mechanical head withdrawal threshold (MHWT) on day 1 after IONI, which persisted until day 11 after IONI (day 1, sham: 58.0 ± 5.8, IONI: 24.4 ± 4.7). Contrastingly, there were no changes in the MHWT after sham treatment throughout the experimental period (Figure 1A). The decrease in MHWT between day 5 and day 11 after IONI was reversed by local administration of high-dose (1.0 × 10^−6^ mol), but not low-dose (1.0 × 10^−8^ mol), OXT to the ligated site of the ION (day 5, vehicle: 35.0 ± 3.7 g, low-dose OXT: 39.6 ± 2.6 g, high-dose OXT: 49.0 ± 3.8 g; day 11, vehicle: 41.8 ± 3.5 g; low-dose OXT: 43.3 ± 3.3 g; high-dose OXT: 61. 0± 3.5 g) (Figure 1B). Further, there was a significant decrease in the heat head withdrawal threshold (HHRT) on day 1 after IONI (day 1, sham: 51.2 ± 0.5 g, IONI: 43.6 ± 1.3 g), which was not reversed by local administration of high-dose (1.0 × 10^−6^ mol) OXT throughout the experimental period (Figure 1C).

### 2.2. OXT Receptor Expression in the TG and ION

**Figure 2:**
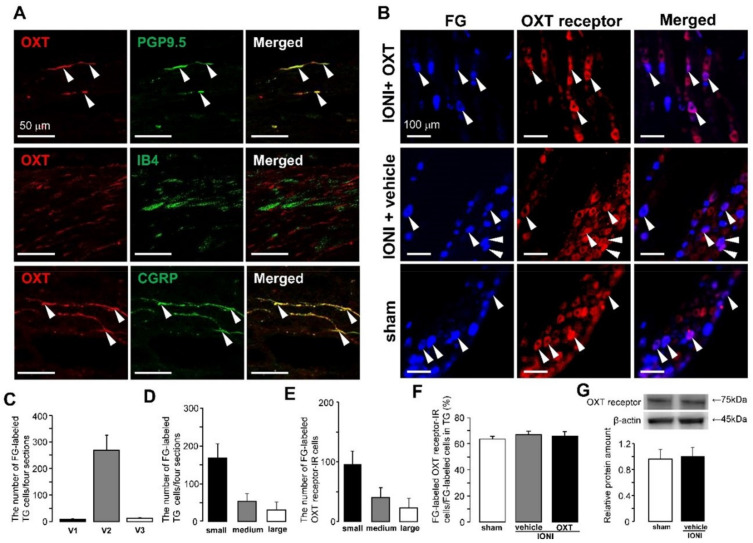
Oxytocin (OXT) receptor expression on day 5 after sham treatment or infraorbital nerve injury (IONI). (**A**) OXT receptor-immunoreactive (IR) nerve afferents that express protein gene product (PGP) 9.5, isolectin B4 (IB4), and calcitonin gene-related peptide (CGRP) in the ION bundle on day 5 after sham treatment. Arrowheads indicate OXT receptor-IR and PGP 9.5-IR, IB4-IR, or CGRP-IR nerve afferents. Scale bar: 50 µm. (**B**) Fluoro-gold (FG)-labeled OXT receptor-IR neurons after sham treatment or IONI with vehicle or OXT administration. Arrowheads indicate FG-labeled OXT receptor-IR neurons. Scale bar: 100 µm. (**C**) The number of FG-labelled neurons in ophthalmic branch division (V1), maxillary branch division (V2), and mandibular branch division (V3) of the trigeminal ganglion (TG) on day 5 after sham treatment. (*n* = 4). (**D**) Size-frequency histogram illustrating the distribution of FG-labeled TG neurons on day 5 after sham treatment. (*n* = 4). (**E**) Size-frequency histogram illustrating the distribution of FG-labeled OXT receptor-IR TG neurons on day 5 after sham treatment. (*n* = 4). (**F**) Mean percentages of FG-labeled OXT receptor-IR TG neurons and FG-labeled TG neurons on day 5 after sham treatment or IONI with vehicle or OXT administration. (sham, *n* = 4; IONI + vehicle, n = 4; IONI + OXT, *n* = 5). (**G**) Relative amounts of OXT receptor in TG. (sham, *n* = 8; IONI + vehicle, *n* = 8).

OXT receptor was expressed in protein gene product (PGP) 9.5, isolectin B4 (IB4), and calcitonin gene-related peptide (CGRP)-positive ION fibers in sham-treated rats (Figure 2A). On day 5 after IONI or sham treatment, OXT receptors were expressed in the fluoro-gold (FG)-labeled cells in the TG (Figure 2B). Most FG-labeled cells were distributed in the maxillary branch division in TG, and predominantly small TG neurons were labeled with FG in sham-treated rats (small: 167.8 ± 38.3, medium: 53.5 ± 20.4, large: 31.5 ± 20.5) (Figure 2C,D). The cell diameter analysis indicated that FG-labeled OXT receptor-immunoreactive (IR) cells mainly belonged to the group with small cell sizes on day 5 after sham treatment (small: 96.3 ± 21.9, medium: 40.5 ± 16.6, large: 23.0 ± 15.9) (Figure 2E). Changes in the number of FG-labeled OXT receptor-IR cells were nonsignificant in the TG after IONI with or without OXT administration or sham treatment (sham: 63.5 ± 2.1%, IONI with OXT: 65.8 ± 3.4%, IONI without OXT: 66.9 ± 2.6%) (Figure 2F). Moreover, the relative amounts of OXT receptor protein in the TG were not changed on day 5 after IONI (sham: 1.0 ± 0.1, IONI with vehicle: 1.0 ± 0.1) (Figure 2G).

### 2.3. Effect of OXT Administration on the TG Neuronal TRPV1 and TRPV4 Expression following IONI

**Figure 3:**
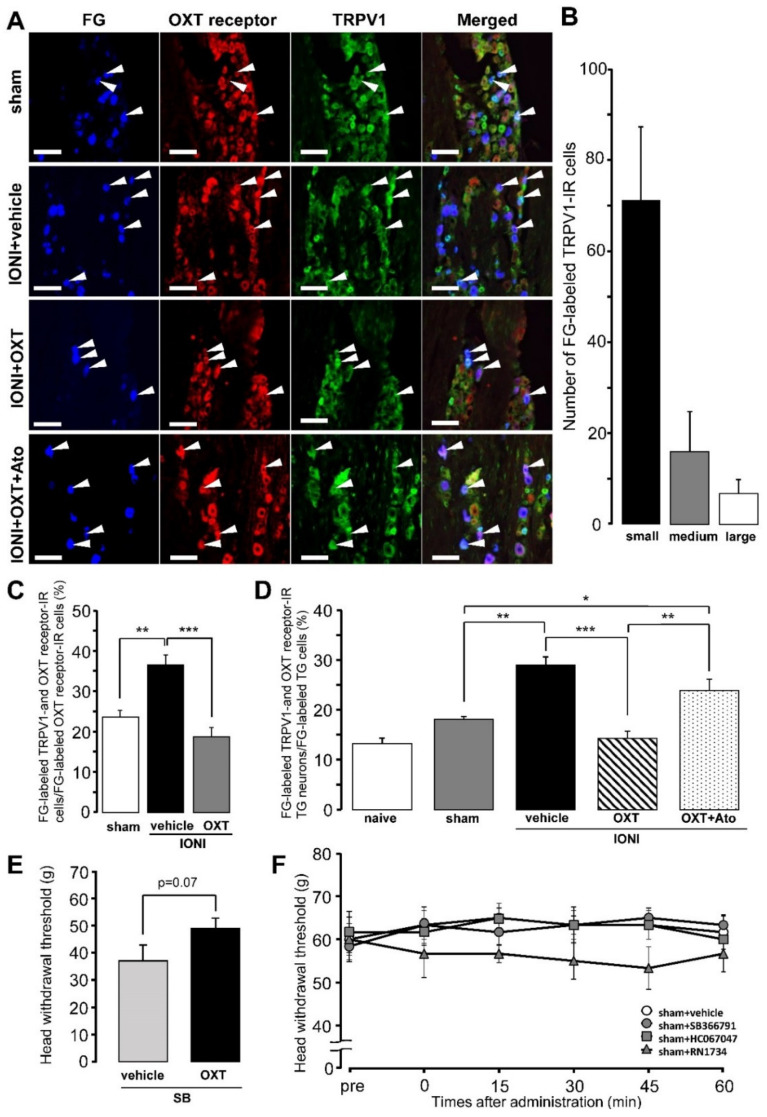
Transient receptor potential vanilloid 1 (TRPV1) and oxytocin (OXT) receptor expression in trigeminal ganglion (TG) neurons on day 5 after sham treatment or infraorbital nerve injury (IONI). (**A**) TRPV1- and OXT receptor-immunoreactive (IR) TG neurons innervating the whisker pad skin on day 5 after sham treatment or IONI with vehicle, OXT, or OXT + atosiban administration. Arrowheads indicate fluoro-gold (FG)-labeled TRPV1- and OXT receptor-IR neurons. Scale bar: 100 µm. (**B**) Number of FG-labeled TRPV1-IR TG neurons on day 5 after sham treatment. (*n* = 4 in each group). (**C**) Mean percentages of FG-labeled TRPV1- and OXT receptor-IR TG neurons out of FG-labeled OXT receptor-IR TG neurons on day 5 after sham or IONI treatment with vehicle or OXT administration. (sham, *n* = 5; IONI + vehicle, *n* = 4; IONI + OXT, *n* = 5). ** *p* < 0.01., *** *p* < 0.001. (**D**) Mean percentages of FG-labeled TRPV1- and OXT receptor-IR TG neurons in FG-labeled TG neurons on day 5 after sham treatment or IONI with vehicle, OXT, or OXT + atosiban administration. (naive, *n* = 5; sham, *n* = 6; IONI + vehicle, *n* = 4; IONI + OXT, *n* = 5; IONI + OXT + Ato, *n* = 6). * *p* < 0.05, ** *p* < 0.01, *** *p* < 0.001. (**E**) The head withdrawal threshold for mechanical stimulation (MHWT) following coadministration of SB366791 and OXT or vehicle to the ligated site of the ION on day 5 after IONI treatment (IONI with SB366791 and vehicle, *n* = 5; IONI with SB366791 and OXT, *n* = 5). (**F**) The MHWT of the whisker pad skin after vehicle, SB366791, HC067047, and RN1734 administration on day 5 after sham treatment (*n* = 6 in each group).

FG-labeled TRPV1-IR TG neurons expressing the OXT receptor were observed in the TG ipsilateral to the site of IONI or sham treatment on day 5 after IONI with or without OXT administration or sham treatment (Figure 3A). Many FG-labeled TRPV1-IR TG neurons were small-diameter cells on day 5 after sham treatment (Figure 3B). IONI caused a significant increase in the percentages of FG-labeled TRPV1- and OXT receptor-IR TG neurons in the FG-labeled OXT receptor-IR TG neuron population, which was completely reversed by OXT administration to the injured-ION bundle (sham: 23.6 ± 1.6%, IONI with vehicle: 36.5 ± 2.4%, IONI with OXT: 18.7 ± 2.3%) (Figure 3C). IONI caused a significant increase in the number of FG-labeled TRPV1-IR TG neurons expressing the OXT receptor. OXT administration to the injured-ION bundle completely reversed the increase in the number of TRPV1-IR TG neurons expressing OXT receptors (Figure 3D). The aforementioned complete recovery was suppressed by the coadministration of OXT with atosiban, which is an OXT receptor antagonist (naive: 13.3 ± 1.0%, sham: 18.1 ± 0.5%, IONI with vehicle: 29.0 ± 1.7%, IONI with OXT: 14.3 ± 1.4%, IONI with OXT and atosiban: 23.8 ± 2.3%). Coadministration of SB366791 and OXT to the ligated site of the ION exhibited a tendency to suppress the recovery of the IONI-induced decrease of MHWT compared to that by administration of SB366791 alone (IONI with SB366791 and vehicle: 37.0 ± 5.8 g, IONI with SB366791 and OXT: 49.0 ± 3.8 g) (Figure 3E). Changes in MHWTs following subcutaneous SB366791, HC067047, and RN1734 administration in the whisker pad skin in sham rats were nonsignificant (Figure 3F).

**Figure 4:**
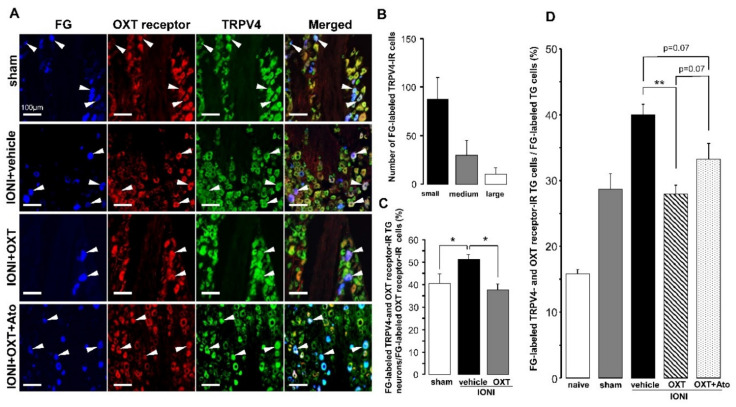
Transient receptor potential vanilloid 4 (TRPV4) and oxytocin (OXT) receptor expression in trigeminal ganglion (TG) neurons on day 5 after infraorbital nerve injury (IONI) treatment. (**A**) TRPV4- and OXT receptor-immunoreactive (IR) TG neurons innervating the whisker pad skin on day 5 after IONI treatment with vehicle, OXT, or OXT + atosiban administration. Arrowheads indicate fluoro-gold (FG)-labeled TRPV4- and OXT receptor-IR neurons. Scale bar: 100 µm. (**B**) Number of FG-labeled TRPV4-IR TG neurons on day 5 after sham treatment. (*n* = 4 in each group). (**C**) Mean percentages of FG-labeled TRPV4- and OXT receptor-IR TG neurons out of FG-labeled OXT receptor-IR TG neurons on day 5 after sham or IONI treatment with vehicle or OXT administration (sham, *n* = 5; IONI + vehicle, *n* = 4; IONI + OXT, *n* = 5). * *p* < 0.05. (**D**) Mean percentages of FG-labeled TRPV4- and OXT receptor-IR TG neurons in FG-labeled TG neurons on day 5 after sham and IONI treatment with vehicle, OXT, or OXT + atosiban administration. (naive, *n* = 5; sham, *n* = 5; IONI + vehicle, *n* = 4; IONI + OXT, *n* = 5; IONI + OXT + Ato, *n* = 6) ** *p* < 0.01.

Furthermore, TRPV4-IR TG neurons expressing OXT receptors that innervated the whisker pad skin were found in the TG ipsilateral to the IONI site on day 5 after IONI with OXT or OXT + atosiban administration (Figure 4A). Many FG-labeled TRPV4-IR TG neurons were small-diameter cells on day 5 after sham treatment (Figure 4B). IONI caused a significant increase in the percentages of FG-labeled TRPV4- and OXT receptor-IR TG neurons in the FG-labeled OXT receptor-IR TG neuron population, which was reversed by OXT administration to the injured-ION bundle (sham: 40.5 ± 4.3%, IONI with vehicle: 51.0 ± 2.2%, IONI with OXT: 37.5 ± 2.7%) (Figure 4C). IONI significantly increased the number of TRPV4-IR TG neurons expressing OXT receptors that innervated the whisker pad skin, which were liable to be recovered by OXT administration to the injured ION bundle site (Figure 4D). The aforementioned recovery exhibited a tendency to be inhibited by the coadministration of atosiban with OXT to the site of the injured ION bundle (naive: 15.8 ± 0.6%, sham: 18.3 ± 0.6%, IONI with vehicle: 40.0 ± 1.6%, IONI with OXT: 28.0 ± 1.3%, IONI with OXT, and atosiban: 33.3 ± 2.4%).

### 2.4. The Inhibitory Effect of TRP Antagonism on IONI-Induced Orofacial Pain Hypersensitivity

**Figure 5:**
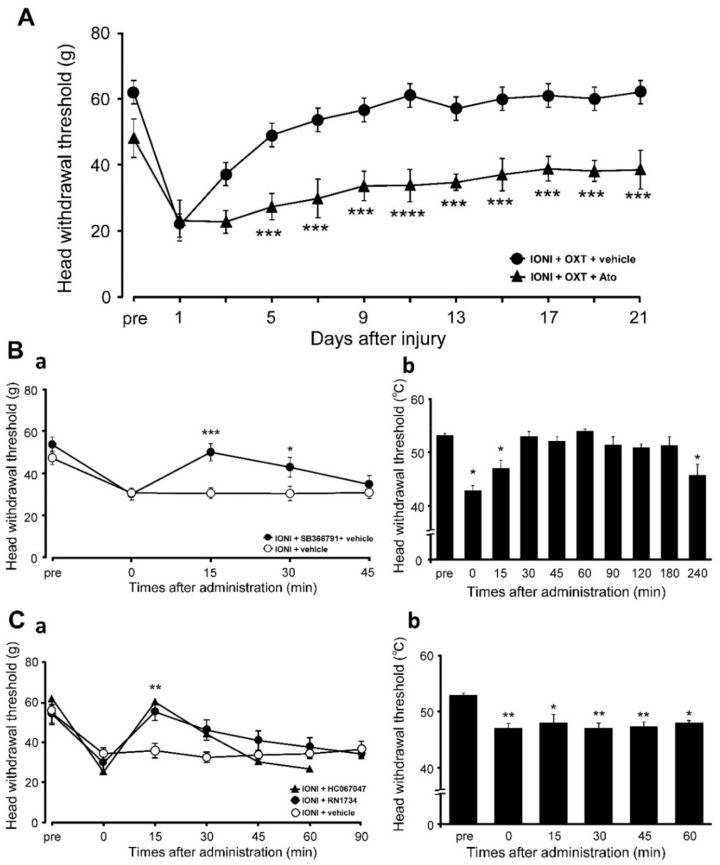
Changes in IONI-induced mechanical hypersensitivities of the whisker pad skin by TRP antagonism. (**A**) The MHWT of the whisker pad skin after IONI with OXT (1.0 × 10^−6^ mol) + vehicle or OXT (1.0 × 10^−6^ mol) + atosiban administration (*n* = 10, in each group). *** *p* < 0.001, **** *p* < 0.0001 (vs. IONI with the OXT + vehicle group). (**Ba**) The MHWT of the whisker pad skin on day 5 after IONI treatment after vehicle or SB366791 administration (*n* = 11, in each group). * *p* < 0.05, *** *p* < 0.001 (vs. the vehicle group). (**Bb**) The HHWT of the whisker pad skin after SB366791 administration on day 5 after IONI treatment (*n* = 5, in each group). * *p* < 0.05 (vs. pre value). (**Ca**) The MHWT of the whisker pad skin after IONI with vehicle, RN1734, or HC067047 administration on day 5 after IONI treatment (IONI + RN1734, *n* = 10; IONI + HC067047, *n* = 6; IONI + vehicle, *n* = 9). ** *p* < 0.01 (vs. IONI + vehicle). (**Cb**) The HHWT of the whisker pad skin after RN1734 administration on day 5 after IONI treatment (*n* = 5, in each group).

The post-IONI decrease in the MHWT was significantly inhibited by local OXT administration to the injured ION bundle site on day 5 after IONI, which persisted throughout the experimental period (Figure 5A). The aforementioned OXT-induced recovery was suppressed by local coadministration with atosiban at 5 days after IONI (day 5, IONI with OXT: 49.0 ± 3.8 g, IONI with OXT, and atosiban: 27.3 ± 3.6 g).

On day 5 after IONI, administration of subcutaneous SB366791, a TRPV1 antagonist, to the whisker pad skin significantly suppressed the MHWT decrease after 15 and 30 min (15 min, IONI with vehicle: 30.6 ± 2.2 g, IONI with SB366791: 50.0 ± 4.0 g) (Figure 5Ba). Additionally, administration of subcutaneous SB366791 suppressed the HHWT decrease from 30 and 180 min (Figure 5Bb). In addition, administration of subcutaneous RN1734 and HC067047, which are TRPV4 antagonists, to the whisker pad skin significantly suppressed the decrease of MHWT at 15 min after administration on day 5 after IONI (15 min, IONI with vehicle: 35.8 ± 3.6 g, IONI with RN1734: 55.0 ± 4.5 g; 15 min, IONI with HC067047: 60.0 ± 5.5 g) (Figure 5Ca). Subcutaneous RN1734 did not change the decreased HHWT on day 5 after IONI (Figure 5Cb).

## 3. Discussion

Traumatic injury to the ION during maxillary oncologic ablation or plastic surgery results in episodes of paresthesia or pain in the ION region [20]. In the present study, IONI caused long-lasting mechanical hypersensitivity and phasic heat hyperalgesia in the ION region, which is consistent with our previous findings [21]. Post-IONI pain hypersensitivities resemble the clinical manifestations of traumatic IONI, which implies that this IONI model is valuable for basic research regarding the mechanisms underlying orofacial traumatic neuropathic pain.

OXT signaling transmitted via its binding to the OXT receptor is considered to be involved in the OXT-induced analgesic effect [6,22]. A recent study reported OXT receptor expression in the sensory neurons of the gelatinous substance of the posterior horn of the spinal cord or dorsal root ganglia [9]. Moreover, the OXT receptor has been identified in the nociceptive neurons of the TG, while orofacial inflammation has been shown to enhance TG neuronal OXT receptor expression [10]. In the present study, OXT receptors were expressed in approximately 30% of TG neurons innervating the whisker pad skin and OXT receptor-expressed TG neurons were mainly small-sized cells, which was consistent with the previous reports [23]. OXT receptors were also expressed in PGP 9.5, IB4, and CGRP-positive nerve afferents in the ION bundle after IONI. Mechanical and heat sensitivity of the whisker pad skin were significantly enhanced immediately after IONI, which persisted for a long time. IONI-induced mechanical, but not heat, hypersensitivity in the whisker pad skin was reversed by constant local administration of OXT to the ligated ION site. OXT signaling was involved in the thermal and mechanical nociception in cultured sensory neurons [24]. The conductivity of sodium or potassium channels in the TG neuron is altered by trigeminal inflammation, resulting in the potentiation of trigeminal pain transmission [25]. Consistent with previous findings, our findings suggest that OXT signaling via the OXT receptor suppresses mechanoreceptor hyperexcitability, which results in OXT-induced relief of orofacial mechanical hypersensitivity after IONI. Moreover, it remains unclear why local OXT administration did not inhibit IONI-induced heat hypersensitivity, which warrants further investigation. However, it is also reported that OXT receptor is localized in the presynaptic or postsynaptic terminals of sensory neurons, which suggests that OXT signaling modulates signal transmission via the sensory neurons [26].

TRPV1 gene expression is mediated in some damaged cells [27]. Notably, repetitive stimulation of the peripheral P2Y1 receptor accelerates upregulation of TRPV1. By extension, inhibiting P2Y1 receptor signaling alleviates heat hypersensitivity and TRPV1 hyperexpression in chronic inflammatory conditions [28]. In this study, the OXT-induced orofacial pain relief was suppressed by TRPV1 antagonism in the whisker pad skin. The TG contains small-sized TRPV1-IR TG neurons expressing OXT receptors that innervate the whisker pad skin. OXT administration to the injured ION bundle site completely inhibited the increase of the number of TRPV1-IR TG neurons expressing OXT receptors. Furthermore, the aforementioned complete recovery was suppressed by OXT receptor antagonism. Taken together, these findings indicate that OXT administration to the site of the injured ION bundle suppresses IONI-induced mechanical hypersensitivity through TRPV1 upregulation in nociceptive TG neurons innervating the whisker pad skin. Furthermore, given that OXT is a known direct TRPV1 agonist, the OXT-induced analgesic effect could be promoted by direct TRPV1 desensitization induced by OXT binding to the nociceptive TG neurons [29]. Through this mechanism, coadministration of TRPV1 antagonist and OXT to the ligated site of the ION may accelerate the recovery of the IONI-induced mechanical pain hypersensitivity compared with that by TRPV1 antagonist alone.

Similar to TRPV1, TRPV4 is a subfamily of TRPV channels and is expressed in the primary sensory neurons [30]. TRPV4 is responsible for mechanical nociception [31,32] and plays an essential role in enhancing C-fiber sensitivity to noxious mechanical stimuli and trigeminal nocifensive behavior. TRPV4 expression is upregulated in the dental sensory nerves during the inflammatory phase [33,34,35]. IONI has been reported to increase TRPV4 expression in the TG neurons [36]. In this study, small-sized TRPV4-IR neurons expressing OXT receptors that innervate the whisker pad skin were also observed in the TG ipsilateral to the IONI. Furthermore, IONI significantly increased the number of TRPV4-IR TG neurons expressing OXT receptors after IONI, which was recovered depending on OXT administration to the ION-injured site. OXT receptor antagonism tends to suppress the increase in the number of TRPV4-IR TG neurons expressing OXT receptors. Furthermore, subcutaneous administration of TRPV4 antagonist into the whisker pad skin suppressed IONI-induced orofacial mechanical hypersensitivity. TRPV4 was expressed in the small-diameter sensory ganglion neurons; moreover, the increased number of TRPV4-positive small-diameter neurons after peripheral nerve injury was reduced [37]. Therefore, OXT signaling could suppress IONI-induced mechanical hypersensitivity by TRPV4 upregulation in nociceptive TG neurons innervating the whisker pad skin.

Peripheral OXT administration suppressed the post-IONI increase in TRPV4-IR TG neurons following IONI; however, OXT receptor antagonism did not completely inhibit the OXT-induced suppression of the increased TRPV4-IR TG neurons. The vasopressin-1A receptor is a known receptor for arginine vasopressin (AVP), which has a similar peptide structure as that of OXT [38]. Furthermore, the OXT receptor shares high sequence homology with vasopressin-1A (V1A) receptor; moreover, both these peptides can activate both the receptors [38]. In addition, OXT signaling via the V1A receptor is expressed in the sensory neurons and mediates OXT-induced analgesia [39]. Subcutaneous AVP administration diminishes nociceptive neuronal activity mediated by Aδ and C fibers, which is interpreted as antinociception [40]. Putative mechanisms of peripheral antinociception by AVP include functional upregulation of the gamma-aminobutyric acid A receptor or inhibition of acid-sensing ion channels (ASICs) expressed in the primary afferents [41,42]. Therefore, OXT administration suppressed TRPV4 upregulation by OXT receptor signaling, as well as the ASIC and nociceptive neuronal activity through V1A receptor signaling. Both effects could contribute to complete suppression of IONI-induced mechanical hypersensitivity. Additionally, the commencement of the inhibitory effect of OXT on mechanical hyperalgesia showed a 5-day delay. It is conceivable that peripheral OXT inhibits the increase in TRPV1 and TRPV4 expression in TG neurons innervating the whisker pad skin; we speculate that it may take several days to synthesize TRPV1 and TRPV4 in TG neurons by OXT signaling. Additionally, it is reported that nerve lesion in branches of the trigeminal nerve led to an increase in the number of TRPV1-positive TG neurons in the uninjured branches of the trigeminal nerve [17]. Therefore, results in this study should be interpreted with caution because it is impossible to establish a proper control condition.

The increase in the number of TRPV1- and TRPV4-positive TG neurons innervating the whisker pad skin contributes to the facilitation of nociceptive processing resulting from IONI as peripheral TRPV1 or TRPV4 antagonism did not induce mechanical hypoalgesia in whisker pad skin in sham rats [36,43]. It is known that TRPV1 activation causes both thermal and mechanical hyperalgesia [44]. Intradermal capsaicin injection has been shown to facilitate dorsal horn neuronal responses due to the input of low-threshold mechanoreceptors and nociceptors [45,46]. TRPV1-positive primary afferent fibers contribute to the increment of ventral root after-discharges induced by application of mechanical stimuli, resulting in mechanical hyperalgesia [47]. Therefore, TRPV1 upregulation by the increased number of TRPV1-positive TG neurons might lead to sensitization of dorsal horn neurons and a decrease in the threshold at which mechanical stimuli detect noxious stimuli. However, TRPV4 can function as a component of an osmotic or mechanical sensor [48]. Isoflurane anesthetics might cause the expression of some pain-related genes in the nociceptor populations in the peripheral nervous system [49]. Although why heat hyperalgesia was not actually induced following IONI is unknown, isoflurane used for light anesthesia for the measurement of HHWT may have had some effect; this is a mechanism that must be studied in the future. From all the results of the experiments, it is probably safe to conclude that TRPV1 and TRPV4 play important roles in the IONI-induced mechanical hypersensitivity.

In summary, peripheral OXT administration attenuated post-IONI orofacial mechanical hypersensitivity. Further, there was a post-IONI increase in the number of TRPV1-IR and TRPV4-IR TG neurons, which was dependent on peripheral OXT signaling. These findings suggest that peripheral OXT inhibits the increase in TRPV1 and TRPV4 expression in TG neurons innervating the whisker pad skin, which attenuated post-IONI orofacial mechanical allodynia. Peripheral OXT administration could be a useful analgesic agent for orofacial neuropathic pain hypersensitivity.

## 4. Materials and Methods

### 4.1. Animals

This study used 205 male Sprague–Dawley rats (200–250 g, Japan SLC, Shizuoka, Japan) maintained on their normal diet under suitable conditions (room temperature: 23 °C; light-dark cycle: every 12 h; ad libitum access to water). All procedures in the study were approved by the Animal Experimentation Committee of Nihon University (AP17D027, 26/1/2018) and complied with the guidelines issued by the International Association for the Study of Pain [50]. All the procedures involved the minimum number of animals and suffering.

### 4.2. Infraorbital Nerve Injury

First, infraorbital nerve injury (IONI) was induced under deep anesthesia with an intraperitoneal (i.p.) injection of the following mixture: butorphanol (2.5 mg/kg; Meiji Seika Pharma, Tokyo, Japan), midazolam (2.0 mg/kg; Sandoz, Tokyo, Japan), and medetomidine (0.15 mg/kg; Zenoaq, Fukushima, Japan) on a warm mat (37 °C) as previously described [21,36,51]. Briefly, the right ION bundle was exposed by detaching the adjacent connective tissue after making a 10-mm right buccal mucosal incision with a scalpel along the gingiva-buccal margin proximal to the first molar. This was followed by tight ligation of one-third of the ION bundle with a 6-0 silk thread (Natsume, Tokyo, Japan) and suturing of the incised wound using another 6-0 silk thread. Rats in the control group underwent sham treatment that was quite indistinguishable except for the IONI.

### 4.3. Assessment of Orofacial Nociception

#### 4.3.1. Mechanical Sensitivity

To assess orofacial mechanical sensitivity, the MHWT of the whisker pad skin was measured as previously described [52]. Briefly, unrestrained animals were initially trained to stick their snouts through a small hole in the cage for mechanical stimulation of the whisker pad skin using von Frey filaments (Touch-Test Sensory Evaluator; North Coast Medical, Morgan Hill, CA, USA) for seven consecutive days.

The whisker pad skin was mechanical stimulated five times at one-minute intervals using the von Frey filaments in ascending order of mechanical intensity (6, 10, 15, 26, 30, 40, 50, 60, and 70 g). The MHWT was defined as the lowest mechanical intensity that produced head withdrawal ≥ 3 times out of the 5 stimuli. After the obtained MHWTs were stable, IONI or sham treatments were performed as aforementioned. Subsequently, the MHWT was assessed under similar and blinded conditions before treatment and every other day for 21 days after treatment. All MHWTs were determined under blinded conditions.

#### 4.3.2. Heat Sensitivity

To assess orofacial heat sensitivity, the animals were primarily anesthetized at an appropriately adjusted level before measuring the HHRT as previously described [53]. Briefly, the animals were maintained at an appropriate anesthesia level through isoflurane inhalation (2%; Mylan, Canonsburg, PA, USA). After aborting the inhalation, the suitable anesthesia depth for HHRT measurement was determined when noxious pinch stimulation (150 g) to the hind paw elicited a complete hind limb withdrawal reflex, as well as when the body temperature, cardiac rhythm, and breathing pattern were appropriate. Graded heat stimulation (35–60 °C, 1 °C/s, cutoff: 60 °C) was directly applied to the whisker pad skin using a contact thermal probe (9 mm^2^) (Intercross, Tokyo, Japan) under a suitable anesthesia depth. The lowest thermal intensity that elicited a head withdrawal reflex was defined as the HHRT. The whisker pad skin underwent heat stimulation (thrice at 5-min intervals) and the mean HHRT was defined as the HHRT for each. All HHRTs were determined under blinded conditions.

### 4.4. Drug Administration

MedGel (MedGEL, Kyoto, Japan), a gelatin-based hydrogel, can form a stable bond with bioactive substances and persistently release them in vivo. In advance, MedGel (6.5 g/rat) was impregnated with OXT (low-dose: 1.0 × 10^−8^ mol, high-dose: 1.0 × 10^−6^ mol, #H-2510, Bachem, Bubendorf, Switzerland) diluted in 0.01 M phosphate-buffered saline (PBS), 20 μL of OXT (1.0 × 10^−6^ mol, diluted in 0.01 M PBS) mixed with OXT receptor antagonist, atosiban (5 μg/mL, #6332, Tocris Bioscience, Bristol, UK), or vehicle (20 μL, 0.01 M PBS). Subsequently, the OXT- or vehicle-containing MedGel was stored overnight at 4 °C. The MedGel volumes, as well as the concentrations of OXT and atosiban, were determined as previously described [54,55,56]. Immediately after IONI under deep anesthesia using an i.p. anesthetizing mixture, OXT- or vehicle-treated MedGel was placed at the ligated site of the ION. Next, the incised skin was closed using 6-0 silk sutures and the MHWTs were measured every other day for 21 days after IONI.

On day 5 after IONI, the TRPV1 antagonist SB366791 (10 μL, 1.6 mg/mL diluted in 50% dimethyl sulfoxide (DMSO) in saline, Tocris Bioscience, Bristol, UK), TRPV4 antagonist, RN1734 (10 μL, 0.4 g/mL diluted in 50% DMSO in saline, Tocris Bioscience), TRPV4 antagonist, HC067047 (10 μL, 30 mg/mL diluted in 50% DMSO in saline, Abcam, Cambridge, UK), or vehicle (10 μL, 50% DMSO in saline) was subcutaneously administered to the whisker pad skin. The concentration of SB366791, RN1734, and HC067047 was determined with reference to previous studies [57,58,59,60]. The MHWTs were measured 0, 15, 30, 45, 60, and 90 min after subcutaneous injection. Additionally, concurrent with IONI, MedGel (6.5 g/rat) impregnated with SB366791 (10 μL, 1.6 mg/mL diluted in 50% DMSO in saline, Tocris Bioscience) mixed with OXT (20 μL, high-dose: 1.0 × 10^−6^ mol, #H-2510, Bachem) diluted in 0.01 M PBS or vehicle (20 μL, 0.01 M PBS) was placed at the ligated site of the ION as described before and the MHWTs were measured on day 5 after IONI. The MHRTs were measured before and 0, 15, 30, 45, and 60 min after subcutaneous vehicle, SB366791, HC067047, and RN1734 injection. Moreover, the HHRTs were measured 0, 15, 30, 45, 60, 90, 120, 180, and 240 min after subcutaneous SB366791 injection and 0, 15, 30, 45, 60, and 90 min after subcutaneous RN1734 injection.

### 4.5. Immunohistochemistry

To identify TG neurons innervating the whisker pad skin, hydroxystilbamidine (Fluoro-Gold [FG]; 10 µL, 4% dissolved in saline) (Fluorochrome, Denver, CO, USA) was subcutaneously injected into the right whisker pad skin immediately after IONI under deep anesthesia using an i.p. anesthetizing mixture. Transcardial perfusion with 4% paraformaldehyde (PFA) solution dissolved in 0.1 M phosphate buffer (pH 7.4) under i.p. deep anesthesia with an i.p. anesthetizing mixture was performed on day 5 following IONI or sham treatment. Subsequently, the TGs, ligated IONs, and sham-treated IONs were dissected and post-fixed in PFA solution for several days at 4 °C. For cryoprotection, the TGs and IONs were immersed in 20% sucrose dissolved in 0.01 M PBS for 12 h. After embedding in Tissue-Tek O.C.T. Compound (Sakura Finetek, Tokyo, Japan), the TGs and IONs were cut at a 10-µm thickness using a cryostat and mounted on Matsunami Adhesive Silane-Coated Superfrost Plus microscope slides (Matsunami, Osaka, Japan). The sections were dried for 12 h at room temperature. For antigen retrieval, the TG and ION sections were incubated in 10% Histo VT One (Nacalai Tesque, Kyoto, Japan) dissolved in distilled water at 70 °C for 20 min. After rinsing with 0.01 M PBS, the TG sections were reacted with anti-OXT receptor goat antibody (1:200; ab87312; Abcam) and anti-TRPV1 rabbit polyclonal antibody (1:400; ACC-030; Alomone Labs, Jerusalem, ISR) or anti-TRPV4 rabbit polyclonal antibody (1:400; ab39260; Abcam, Cambridge, UK) diluted in 0.01 M PBS with 1% skimmed milk and 0.1% Triton-X (Merck, Darmstadt, Germany) at 4 °C for 2 days. The ION sections were reacted with anti-OXT receptor antibody (1:200; ab87312; Abcam) and anti-PGP 9.5 mouse monoclonal antibody (1:500; Abcam) or isolectin IB4 from Griffonia simplicifolia, Alexa Fluor 488 conjugate (1:1000; Thermo Fisher Scientific, Waltham, MA) or anti-calcitonin gene-related peptide mouse monoclonal antibody (Merck) as described previously. After rinsing with 0.01 M PBS, the TG sections were reacted with anti-rabbit donkey Alexa Fluor 488 IgG (1:100; ab150077; Abcam) and anti-goat donkey Alexa Fluor 594 IgG (1:100; ab150116; Abcam) followed by anti-goat donkey Alexa Fluor 594 IgG (1:100; ab150116; Abcam) for 3 h at room temperature. Subsequently, the TG and ION sections were coverslipped in PermaFluor mounting medium (Thermo Fisher Scientific). Using a BZ-9000 system (Keyence, Osaka, Japan) equipped with the appropriate filters, the image (3812 × 2743 μm^2^) containing the entire TG was obtained. Four TG sections were used in the subsequent cell counting. FG-labeled OXT receptor/TRPV1-IR neurons and FG-labeled OXT receptor/TRPV4-IR TG neurons were identified in the TG sections; furthermore, OXT receptor-IR nerve fibers were identified in the ION sections. In the TG sections photographed under the same conditions (exposure time, FG: 1/1.2 s; OXT receptor: 1/1.2 s; TRPV1: 1/1.8 s; TRPV4: 1/1.8 s), only a fluorescence intensity of twice or more compared to the average background signal was defined as IR. No specific immunoreactivity was observed in the absence of primary antibodies. The proportions of FG-labeled OXT-IR TG neurons, FG-labeled OXT receptor-TRPV1-IR TG neurons, and FG-labeled OXT receptor-TRPV4-IR TG neurons were calculated using the following formulas: 100 × total number of FG-labeled OXT receptor-IR neurons/total number of FG-labeled neurons, 100 × total number of FG-labeled OXT receptor-TRPV1-IR neurons/total number of FG-labeled neurons, 100 × total number of FG-labeled OXT receptor-TRPV4-IR neurons/total number of FG-labeled neurons, and 100 × total number of FG-labeled OXT receptor-TRPV1-IR or OXT receptor-TRPV4-IR neurons/total number of FG-labeled OXT receptor-IR neurons with reference to past reports [21,36]. Regarding the FG-labeled and FG-labeled TRPV1-, TRPV4-, and OXT receptor-IR TG neurons, the number of each group of cells classified by IR area (<599 µm^2^, 600–999 µm^2^, >1000 µm^2^) was calculated according to a previous report [36]. Furthermore, the number of FG-labeled TG neurons was counted according to the three branches of the trigeminal nerve previously reported [61].

### 4.6. Western Blotting

On day 5 after IONI or sham treatment, the rats were perfused with physiological saline under deep i.p. anesthesia with the above-described solution. The tissue including the injured or sham-treated ION and TG was removed immediately and homogenized in ice-cold lysis buffer (137 mM NaCl; 20 mM Tris-HCl, pH 8.0; 1% NP40; 10% glycerol; 1 mM phenylmethylsulfonyl fluoride; 10 μg/mL aprotinin; 1 g/mL leupeptin; and 0.05 mM sodium vanadate). The homogenate was centrifuged, and the supernatant was collected. The protein concentration of the supernatant was determined using a protein assay kit (Bio-Rad, Hercules, CA, USA). Supernatants were heat-denatured in Laemmli sample buffer solution (Bio-Rad), and the samples with protein concentration adjusted to 30 μg were subjected to electrophoresis on 10% sodium dodecyl sulfate-polyacrylamide gel electrophoresis for protein separation. The samples were transferred to a polyvinylidene difluoride membrane (Trans-Blot Turbo Transfer Pack; Bio-Rad). The membrane was rinsed with Tris-buffered saline mixed with 0.1% Tween 20 (TBST) and incubated in 3% bovine serum albumin (BSA; Bovogen, Essendon, Australia). The membrane was then incubated overnight at 4 °C with anti-OXT receptor antibody (1:300; ab87312; Abcam) diluted in TBST with 3% BSA. Then, it was incubated with horseradish peroxidase-conjugated rabbit anti-goat antibody (Jackson Immuno Research, West Grove, PA, USA) for 2 h at room temperature. Protein-bound antibodies were detected using Western Lightning ELC Pro (PerkinElmer, Waltham, MA, USA) and visualized using a ChemiDoc MP system (Bio-Rad). Using β-actin antibody (1:200; Sc-69879, Santa Cruz, CA, USA), following removal of bound protein by a stripping reagent (Thermo Scientific), protein levels were normalized to β-actin.

### 4.7. Statistical Analysis

MHWT and HHWT data are presented as mean ± standard error (SEM). Two-way analysis of variance (ANOVA) with repeated measures followed by Tukey’s multiple comparison test or one-way ANOVA followed by Dunnett’s multiple comparison test were performed to analyze changes in the MHWT and HHWT. Furthermore, data regarding changes in the immunohistochemical properties of TG neurons are presented as the mean ± SEM. One-way ANOVA followed by Tukey’s or Holm-Sidak multiple comparison test was performed for TG neuronal immunohistochemical analysis. A *p*-value of <0.05 was considered statistically significant.

## Abbreviations

IONI	Infraorbital nerve injury
OXT	Oxytocin
Ato	Atosiban
TG	Trigeminal ganglion
MHWT	Mechanical head-withdrawal threshold
HHRT	Heat head-withdrawal threshold
FG	FluoroGold
TRPV	Transient receptor potential vanilloid
PGP	Protein gene product
IB4	Isolectin B4
CGRP	Calcitonin gene-related peptide
ASICs	Acid-sensing ion channels
